# Newly Diagnosed Type 1 Diabetes in an Elderly Patient

**DOI:** 10.7759/cureus.43646

**Published:** 2023-08-17

**Authors:** Inês Cosme, Ema Nobre, Maria João Bugalho

**Affiliations:** 1 Endocrinology, Diabetes and Metabolism, Centro Hospitalar Universitário Lisboa Norte, Lisbon, PRT; 2 Clínica Universitária de Endocrinologia, Faculdade de Medicina, Universidade de Lisboa, Lisbon, PRT

**Keywords:** elderly onset, diabetic keto acidosis, type 1 diabetes, geriatric diabetes, diabetes antibodies

## Abstract

Type 1 diabetes (T1D) is typically diagnosed in young people; however, it can appear at any age. Its incidence in adulthood is not as well-known as in childhood, particularly if it is diagnosed in geriatric age. T1D diagnosed in adulthood can be explained by the development of antibodies in adulthood or also by the existence of slow-disease progressors.

A 71-year-old normal-weight woman presented to the Emergency Department complaining of polyuria, polydipsia, and tiredness. She was identified with hyperglycemia (450mg/dL) and high blood and urine ketone bodies. Her arterial gasometry revealed mild metabolic ketoacidosis. Further laboratory work-up was remarkable for positive anti-GAD and anti-ICA antibodies and her HbA1c was 14.1%. The diagnosis of T1D was established. A urinary infection was also identified.

The patient’s symptoms in association with metabolic ketoacidosis, in the presence of high titers of more than one positive T1D-related antibody, have helped us to diagnose T1D in this elderly woman. A prompt diagnosis enabled us to establish adequate diabetes treatment. The urinary infection was probably a trigger to the symptomatic phase of diabetes.

T1D can be diagnosed at any age, even in elderly patients. A prompt T1D diagnosis can avoid the misdiagnosis of type 2 diabetes (T2D), enabling the beginning of correct medication earlier.

## Introduction

Type 1 diabetes (T1D) results from the destruction of insulin-secreting islet cells by an immune-mediated process, which may be induced or promoted by genetic or environmental factors [1].

The incidence of T1D is higher in children; however, its onset can occur at any age [2]. The epidemiology of adult-onset T1D is not well-characterized as it is in childhood. This may be explained by several reasons: historic focus on T1D as a common children's disease, challenges in distinguishing adult-onset T1D from type 2 diabetes (T2D), and fewer registries of T1D incidence in adulthood, compared with childhood records [3].

Recognition of adult-onset T1D is important because, if misclassified as T2D, it may lead to inappropriate management and the occurrence of morbidity [3]. 

The incidence of adult-onset T1D is reported to be similar to childhood-onset incidence, i.e., countries with higher rates in childhood tend to have higher rates in adulthood [4]. Despite this, the exact incidence of T1D in adulthood and mainly in elderly people remains unclear [5]. 

Geriatric T1D is anticipated to become more common over the next decade [6]. In one large American T1D registry (T1D Exchange), including 22697 participants, 15% were T1D patients diagnosed with > 50 years [6]. Moreover, approximately 30 cases of T1D onset, including fulminant cases, have been reported since 1997, in patients ≥ 75 years [7].

Diabetes can be presented with an acute complication, particularly in T1D cases. The frequency of diabetic ketoacidosis (DKA) among adults, with T1D diagnosis, is unclear but is believed to be lower than in childhood onset [8], because patients who have an adult-onset of T1D have a residual β-cell function and insulin production with persistent measurable C-peptide and, for these reasons, better glycemic control [9]. 

In adult-onset T1D, multiple positive diabetes autoantibodies are less prevalent with the increase of the age at diagnosis, with glutamic acid decarboxylase (GAD) antibody being the most frequent positive autoantibody [10]. In a recent analysis, the five-year rate of progression to diabetes, in adults with multiple positive autoantibodies, was 15% [10].

The SNAIL study (Slow or Nonprogressive Autoimmunity to the Islets of Langerhans study) identified patients, named slow progressors, who were free of diabetes symptoms for more than 10 years, despite having multiple positive autoantibodies [11]. Despite these data, slow progressors may not represent all autoimmune adult-onset T1D cases, indicating that probably autoantibodies must arise at all ages [10].

## Case presentation

A 71-year-old woman presented to the Emergency Department of our hospital complaining about polyuria, polydipsia, and tiredness for a two-week duration. The patient denied weight loss.

Her medical history was remarkable for hypertension diagnosed at the age of 65, chronic ferropenic anemia, allergic rhinosinusitis, and asthma. She was medicated with losartan 100 mg daily, nebivolol 5 mg daily, an oral iron supplement daily, and inhaled salmeterol/fluticasone propionate twice daily.

The patient previously did not have diabetes, neither was they on antidiabetic medications, as well as had not any relatives with diabetes.

On admission, her physical examination was unremarkable, except for dehydrated skin and mucous membranes. Her vital signs were a body temperature of 36.3 °C, a blood pressure of 116/67 mmHg, a heart rate of 81 beats/min, and a respiratory rate of 14 breaths/min on room air. Her BMI was 24.1 Kg/m^2^ (height 164 cm; weight 65 Kg). Her capillary blood glucose was 450 mg/dL, and her urine and blood ketone bodies were positive. The arterial gasometry showed metabolic acidosis (Table 1). A saline infusion and a continuous intravenous infusion of fast-acting insulin according to glycemia were started.

**Table 1:** Patient's initial laboratory evaluation The reference ranges presented are the ranges used in our hospital. GAD: Glutamic acid decarboxylase; IA-2:  tyrosine phosphatase-related islet antigen 2; ICA: islet cell antibody

Arterial blood gas	Result	Reference range
pH	7.32	7.35-7.45
pCO_2 (_mmHg)	23	35-45
pO_2 (_mmHg)	99	75-100
HCO_3_^- ^(mmol/L)	15	22-26
Anion gap (mmol/L)	12	8-16
Glycemia (mg/dL)	450	-
K^+ ^(mmol/L)	3.1	3.5-4.5
Diabetes evaluation		
Anti-GAD (U/mL)	>280	≤17
Anti-ICA (U/mL)	222.7	<28
Anti-IA-2 (U/mL)	7.3	≤28
Anti-insulin (U/mL)	5.1	<20
C-peptide (ng/dL)	1.4	2.1-4.3
Blood cell count		
Hemoglobin (g/dL)	13.9	12-15.3
White blood cells (x10^9^/L)	5.7	4-11
Platelets (x10^9^/L)	123	150-450
Blood chemistry		
AST (U/L)	22	0-32
ALT (U/L)	12	0-33
ALP (U/L)	80	35-105
Amylase (U/L)	46	13-53
BUN mg/dL	119	16-49
Creatinine (mg/dL)	1.41	0.5-0.9
Na^+ ^(mmol/L)	144	135-145
K^+ ^(mmol/L)	3	3.5-4.5
Cl^- ^(mmol/L)	100	98-107

Concerning a more complete diabetes laboratory evaluation, other tests were performed. Their results were: HbA1C 14.1%, positive anti-GAD and anti-ICA (islet cell antibody) antibodies, and a serum C-peptide of 1.4 ng/mL (table 1).

An abdominal CT scan was also done which excluded any pancreatic abnormality, including a pancreatic tumor or acute/chronic pancreatitis. The urine analysis was compatible with acute cystitis (*S.agalactiae* was isolated in the urine culture). 

Based on the markedly elevated titers of anti-GAD and anti-ICA antibodies, in association with marked hyperglycemia and metabolic acidosis with positive ketone bodies, the diagnosis of an acute onset of autoimmune T1D was established.

After correcting the DKA, the patient was hospitalized and started on a sliding scale of fast-acting insulin before each meal and long-acting daily insulin. During her hospital admission, the patient learned how to administer insulin and self-evaluate glucose levels. The cystitis was also treated with an adequate antibiotic chosen according to the antibiotic sensitivity test. The patient was medicated with cefuroxime.

Currently, she is medicated with very long-acting insulin (degludec) in a dose of 8 IU daily and a sliding scale of fast-acting insulin before the major meals, to which she adheres properly and has adequate metabolic control. The patient's recent HbA1C is 6.6% and the time in range is 85%.

## Discussion

We report a case of acute onset of T1D, presented with hyperglycemic symptoms and DKA, in a 71-year-old normal-weight woman, who had significantly elevated titers of two antibodies.

Although islet autoantibodies can also be positive in the general population, high titers or the presence of more than one positive antibody increases the likelihood of T1D [12,13]. 

In this case, the authors consider that antibodies with higher titers in association with the clinical presentation, the DKA episode, in a patient with an adequate body mass index, can establish the T1D diagnosis.

According to Leslie et al., it is recommended to measure the islet antibodies and C-peptide in older people with features suggesting T1D [14]. The measurement of serum C-peptide, paired with blood glucose in the same sample, provides an estimate of endogenous insulin production and may be useful to estimate the duration of diabetes. The cut-off of serum C-peptide to differentiate T1D from T2D at diagnosis has not been defined yet; however, C-peptide levels >1.8 ng/mL are more suggestive of T2D. Adults often have higher C-peptide levels at diagnosis and a slower decline in β-cell function over time, so the C-peptide at diagnosis is higher in adults than in child patients [11]. Our patient had a serum C-peptide level of 1.4 ng/mL, which favored the diagnosis of T1D and showed a residual pancreatic function.

As stated by Gao et al., even in subjects with T1D with high titers of antibodies, insulin secretory capacity can be preserved in cases of slowly progressive T1D [12]. We consider that this patient is probably a case of slow progressive T1D with a β-cell function preserved during a long period, despite the high autoantibodies’ titers. Probably, the urinary disease was an abrupt event that promoted the transition to the diabetes clinical phase, favoring the occurrence of DKA. Another possible explanation of the physiopathology of this case [10], instead of the slow progressive T1D, is that autoantibodies developed during a later time in adulthood.

A prompt T1D diagnosis in elderly people is essential because diabetes misclassification occurs in up to 40% of adults >30 years, which may result in inappropriate management [13].

The restoration of the endogenous insulin secretion capacity, which may explain the reduced necessary amount of daily insulin after discharge, was considered to be the result of the release of glucose toxicity or being part of the honeymoon period. The honeymoon period, which appears shortly after the onset of T1D, can persist for a few months to years. In adults, there are no reports about the duration of the honeymoon period. During this period, some patients regain β-cell activity transiently, their insulin requirements are minimal to maintain adequate glycemic control, and, in a few cases, it is even possible to maintain a normal/near normal blood glucose level with reduced or without insulin [7]. 

Another important aspect to address in older patients is that insulin treatment strategies and delivery approaches must be individualized and differentiated between healthy older adults and those with frailty, severe comorbidities, and limited life expectancy [15]. In this case, as we were facing a patient without severe comorbidities, who is autonomous and independent in daily activities, we prescribed a scheme of insulin self-injections together with self-monitored blood glucose levels.

## Conclusions

This clinical case highlights that T1D can be presented in geriatric patients. With the aging of the population, cases of T1D diagnosis will increase, so it is important to make a prompt diagnosis in order to start insulin therapy earlier. Probably, the C-peptide value of this patient may explain a residual insulin secretory capacity that maintained glucose control stable for several years, despite the presence of positive antibodies. T1D treatment in older patients should be adapted to their comorbidities and lifestyle.
